# Computational Analysis of Amine Functionalization in Zwitterionized Polyether Sulfone Dialysis Membranes

**DOI:** 10.3390/membranes14110226

**Published:** 2024-10-29

**Authors:** Simin Nazari, Arash Mollahosseini, Amira Abdelrasoul

**Affiliations:** 1 Division of Biomedical Engineering, University of Saskatchewan, 57 Campus Drive, Saskatoon, SK S7N 5A9, Canada; 2 Department of Chemical and Biological Engineering, University of Saskatchewan, 57 Campus Drive, Saskatoon, SK S7N 5A9, Canada; 3 National Institute of Nanotechnology, National Research Council Canada, 11421 Saskatchewan Dr NW, Edmonton, AB T6G 2M9, Canada

**Keywords:** hemodialysis membrane, amine functionalization, water stability, affinity of interactions, hemocompatibility

## Abstract

Hemodialysis is a critical treatment for patients with end-stage renal disease (ESRD) who lack kidney transplant options. The compatibility of hemodialysis membranes is vital, as incompatibility can trigger inflammation, coagulation, and immune responses, potentially increasing morbidity and mortality among patients with ESRD. This study employed molecular dynamics simulation (MDS) and molecular docking to assess the hemocompatible properties of Polyether Sulfone (PES) membranes modified via two distinct amine functionalization techniques. The molecular docking results demonstrated that side amine functionalization exhibited a lower affinity energy (−7.6) for fibrinogen compared to the middle amine functionalization (−8.2), suggesting enhanced antifouling properties and superior hemocompatibility. Additionally, side amine functionalization formed hydrogen bonds with four amino acids, enhancing its resistance to protein adhesion compared to three amino acids in the middle amine structure. Furthermore, the molecular dynamics simulations revealed differences in water mobility, with the side amine functionalized membranes showing a lower mobility value (9.74 × 10^−7^) than those treated with the middle amine method (9.85 × 10^−7^), indicating higher water stability and potentially better patient outcomes. This study’s findings contribute to the design of more efficient and safer hemodialysis treatments by optimizing membrane materials.

## 1. Introduction

Membrane separation has its place among the high-tech separation processes that serve fields such as the water treatment, fuel processing, gas separation, biomedical, and food fields. Polymeric membranes are easy to fabricate. The raw materials have a significant range of variety, and a range of characteristics are available for the membrane separation scientists to pick from. Several desired characteristics of the polymeric membranes, however, will be added to the polymeric matrix by future physicochemical treatments. The functionalization of polymeric substances either before fabrication of membranes or after an initial separative membrane is possible, offering several added properties and control over the performance of the final product.

Polyether sulfone (PES) is a well-known synthetic polymer with distinctive features including exceptional thermal, mechanical, and hydrolytic strength in both hot and wet conditions [1]. PES is the main compound used in dialysis membranes [2,3]. Additionally, PES is known to be hydrophobic in nature [4,5]. This results in a high degree of hemoincompatibility (protein adsorption on the membrane surface, clotting of blood) as well as loss of membrane performance within the dialysis process. Accordingly, modification approaches are necessary for PES membranes.

Several reviews have covered the functionalization of materials in bioengineering applications to control cell–material interaction [6,7,8]. A broad spectrum of modification approaches are available for dialysis applications. The latest advances in dialysis membrane enhancements and zwitterionization techniques have been reviewed by Mollahosseini et al. The immobilization of a biomimetic structure, zwitterion, amino acid, or any other surface enhancer involves an initial surface treatment [9,10]. The interfacial modification of PES membranes is commonly achieved by an initial step of sulfone, carboxyl, hydroxyl, or amine functionalization [10,11].

**Figure 1:**
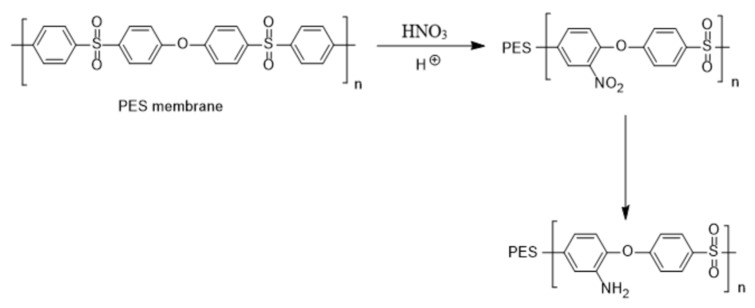
Amine functionalization of PES membrane using acid washing and reduction reactions with HNO_3_ (Scheme created by our research group).

**Figure 2:**
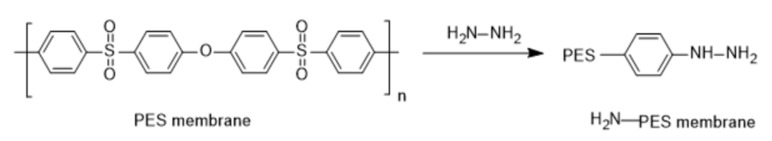
Aminolysis of PES membrane to locate amine functional groups on the membrane surface (Scheme created by our research group).

The mine functionalization (amination) of PES has been reviewed recently [12]. Electrophilic amine groups are particularly efficient in attaching to polymer chains due to their high reactivity [13]. A common process for such amine functionalization is acid washing of the membranes followed by a reduction reaction, which turns NO_2_ to NH_2_ amine functional groups (Figure 1) [13]. It is commonly assumed that modifying PES to introduce NH_2_ is challenging; however, the contrary is true. The presence of strong electron-withdrawing groups (sulfonyl) makes phenylene rings highly electron-deficient. Accordingly, the nucleophilic-substitution reaction on the aryl ether bonds is enhanced. An instance of this approach is performed by aminolysis of PES membrane as shown in Figure 2 [14,15,16].

The two feasible approaches for PES amination result in different locations of the amine groups. Our team has focused on the modification of PES membranes through zwitterionization approaches. Amine amination is the first step in several chemical reactions that we have designed. Each approach to amination could result in a slightly different final chemical structure as the product. Accordingly, it was important for us to assess the two approaches. A comparison of the two approaches was conducted through the simulation of the final zwitterionized products, one with a middle amine (according to the first approach shown in Figure 1) and one with a side amine (according to the second approach reflected in Figure 2).

This study evaluated two aminated PES membranes through molecular docking and molecular dynamics simulations. The purpose of this article is to analyze and delineate the potential differences between the two functionalization strategies using both molecular dynamics (MD) simulations and molecular docking techniques. The objectives were (i) to compare the stability of the water-aminated PES systems; (ii) to assess the hydrophilicity of the simulated zwitterionized polymers by examining hydrogen bonding profiles; and (iii) to investigate the binding energy between the two amine-modified membranes and fibrinogen using molecular docking, highlighting the implications for hemocompatibility.

## 2. Computational Methods

### 2.1. Molecular Docking

**Figure 3:**
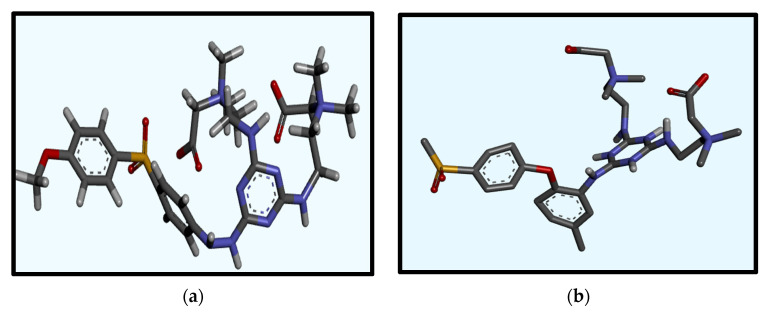
Schematic representation of PES-S-TA-CB (**a**) and PES-M-TA-CB structures (**b**).

Fibrinogen, a critical plasma protein, plays a pivotal role in blood clotting and vascular injury responses. Understanding how fibrinogen interacts with biomaterial surfaces, especially at the interface, is essential for improving the design of membranes with optimal hemocompatibility. These interactions are crucial in activating the coagulation cascade and facilitating platelet adhesion, both key factors in the development of thrombosis. Insight into how fibrinogen binds to artificial surfaces aids in the engineering of materials designed to either minimize unwanted clot formation or enhance blood compatibility. This is crucial for the safety and efficacy of blood-contacting medical devices such as vascular grafts, stents, and dialysis membranes [15,16]. Molecular docking is employed to elucidate molecular interactions and to simulate these interactions computationally. This pivotal technique is fundamental for the design of biomedical devices and structure-based drug development, providing precise predictions of the interactions between small molecules (ligands) and specific protein receptors (fibrinogen). We acquired the 3D X-ray structure of fibrinogen (FB, PDB code: 3GHG) [17] from the RCSB Protein Data Bank for our docking studies. Using ChemDraw software and Chem3D Ultra (ChemDraw Ultra 12.0, PerkinElmer Informatics, Inc., Waltham, MA, USA), we sketched and optimized the monomer structures, which were then converted to PDB format using PyMOL 2.5.5 (Designed by: Schrödinger, LLC, New York, NY, USA), offering advanced 3D visualization capabilities. Our docking analysis involved simulations using AutoDock Vina version 4.0 (Developed by the Scripps Research Institute, La Jolla, CA, USA). We prepared the protein structure by removing water molecules, merging nonpolar hydrogens, and adding Kollman charges with AutoDock Tools 4.2.6. (The Scripps Research Institute, La Jolla, CA, USA). Active sites were identified within a docking box with dimensions of 40 × 40 × 40, centered such that X = 56.94Å, Y = 3.209Å, Z = −54.55Å aligned directly with the central active site of the fibrinogen protein. Residues containing atoms larger than 7 Å were excluded to enhance docking efficiency. The Lamarckian genetic algorithm (LGA) was employed with a set run of 50 iterations to ensure the reliability of the data [18,19]. Interaction analysis involved measuring intermolecular energy to analyze the interactions between the docked proteins and zwitterionic ligands, thereby assessing the binding patterns. The structure of the ligand is depicted in Figure 3. For visualization, we displayed all energy-minimized conformers using tools such as ChemDraw, PyMOL, and Discovery Studio Visualizer 2024 Client (BIOVIA, Dassault Systèmes, San Diego, CA, USA).

### 2.2. Molecular Dynamics Simulation

The Dreiding force field potential was used for describing the correlation between the atoms in the structures [20]. Table 1, Table 2 and Table 3 summarize the parameters. The chosen potential is a proper force field to model both polymeric and biological structures. The correlations between the atoms could be classified into two categories: bond and non-bond interactions. Bond interactions cover bond stretching, bond angles, and dihedral angles, while non-bond interactions include van der Waals and electrostatic interactions.

The Dreiding force field was employed to describe the molecular interactions within our simulations, focusing on general principles such as bond stiffness, equilibrium conditions of the bonds and angles, and interaction strengths. This modeling approach was essential for capturing the detailed behaviors of atoms within the PES membrane structures, providing insights into their hemocompatibility. These interactions, both bonding and non-bonding, are crucial for understanding the membrane’s performance in hemodialysis applications [21,22,23].

**Figure 4:**
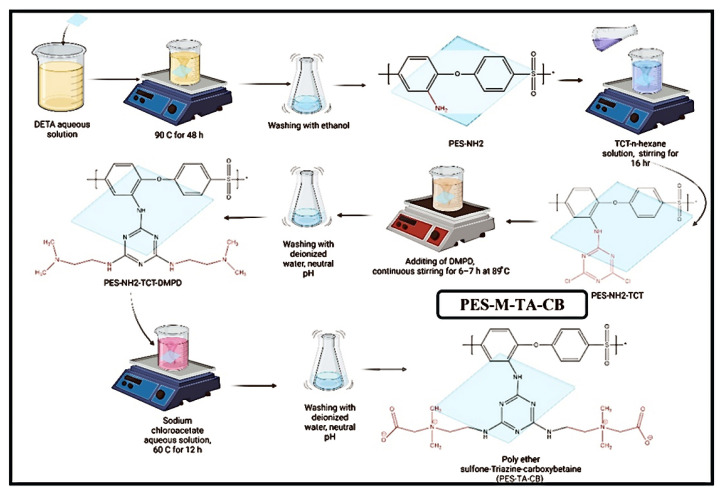
Chemical route to immobilize TA-CB on PES membrane using middle amine functionalization. Chemical route created by our research group, Adapted from our patent WO2024092356A1 (Abdelrasoul et al., 2024) [16].

**Figure 5:**
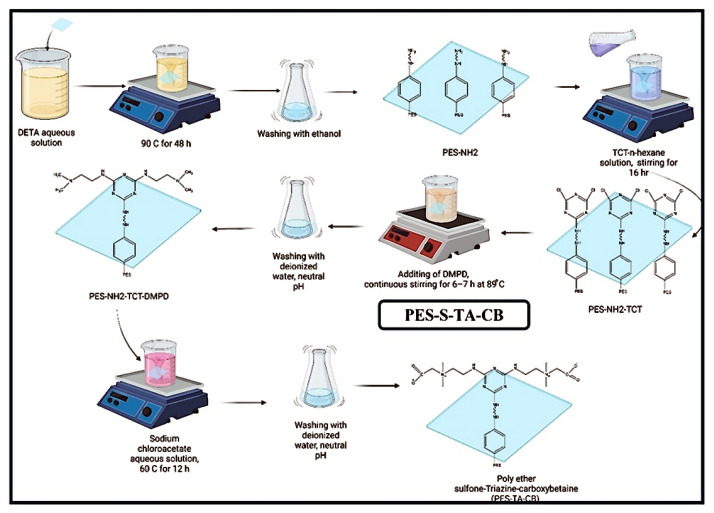
Chemical route to immobilize TA-CB on PES membrane using side amine functionalization. Chemical route created by our research group, Adapted from our patent WO2024092356A1 (Abdelrasoul et al., 2024) [16].

Table 4 categorizes the named chemical structures that were created and minimized using AVOGADRO software (Version 1.99.0) [24] (chemical routes to feasibly immobilize triazine carboxybetaine (TA-CB) structures on PES membranes through middle and side amine functional groups are illustrated in Figure 4 and Figure 5, respectively). VMD (Version 1.9.4.) and OVITO software (Version 3.8.5, Alexander Stukowski, Darmstadt, Germany)were used for visualization purposes [25,26]. LAMMPS software (Version 3) was used for MD simulations [27]. Simulation boxes were created using PACKMOL software (Version 20.14.0) [28]. The size and the number of atoms in each simulation are included in Table 4. Periodic boundary conditions were assumed for all the directions, and the simulations were performed using real units. The performed MD simulations were conducted at 298 K using the Langevin thermostat and NVE ensemble.

### 2.3. Assessing Water Mobility Using Molecular Dynamics Simulation

To evaluate the potential of the newly proposed membrane structures in reducing water molecule mobility, we conducted mobility simulations using MSD analysis. This approach was instrumental in understanding how modifications to membrane structures impact the diffusion behavior of water molecules, which is critical for assessing hemocompatibility and overall membrane performance.

In our study, we used PES-M-TA-CB and PES-S-TA-CB structures to create a membrane model. To perform this, 50 monomers of each structure were located in a slab using PACKMOL software. One thousand water molecules were located on the sides of the slab to create the hydrated simulation box. Transferable intermolecular potential with 3 points (TIP3P) was used as the model for water molecules. Simulations were performed to calculate the mobility function of water molecules according to the following equation [29]:

(1) $$C\left(∆t,Z\right)=\frac{\langle {{(r}_{i}\left(t_{0}+∆t\right)-r_{i}\left(t_{0}\right))}^{2}\rangle z}{MSD\;of\;pure\;water}$$

where the term in < > is the average mean square displacement ensemble, *r_i* is the location of the molecules, r_i (t_0) is the location of structure i at time t0, r_i (t_0 + ∆t) is the location of structure i after ∆t, Z is the height of the structures, and MSD is the mean square displacement of the structures. The simulations were performed at room temperature using the NVE ensemble and Langevin thermostat. Simulations were run in 0.01 fs time steps for a total run time of 100 ps.

Rationale for using MSD:

- Insight into molecular dynamics: MSD offers a detailed perspective on the diffusion and interaction of water molecules with the membrane, revealing how structural modifications influence mobility.
- Comparison to pure water: By normalizing the MSD of the modified structures to that of pure water, we could quantitatively assess the effectiveness of the proposed modifications in a restricted water movement.
- Critical for hemocompatibility: Reduced mobility is often associated with improved hemocompatibility, making MSD a crucial metric for evaluating the potential application of the membranes in biomedical settings.
- Complementary to structural analysis: While RMSD focuses on structural deviations, MSD enriched our analysis by focusing on dynamic properties, providing a comprehensive view of the material’s behavior.

## 3. Results and Discussion

### 3.1. Evaluating Membrane Models’ Interactions with Human Serum Fibrinogen

Computational techniques like molecular docking have become indispensable in biomedical device design and pharmaceutical development. When designing biomaterials that interact with blood, preventing adverse interactions with blood components and mitigating risks like activation and degradation are paramount. Molecular docking offers a robust framework to comprehensively assess potential negative interactions between newly engineered materials and blood components, thereby enhancing safety and efficacy.

In this study, we focused on fibrinogen, a crucial protein involved in both protein adsorption at biomaterial–blood interfaces and in coagulation and thrombosis mechanisms. Our molecular docking analysis specifically focused on amine-modified membranes to evaluate their interactions with fibrinogen and assess hemocompatibility. By analyzing the preferred binding sites, conformations, orientations, and binding energies, we aimed to identify structural characteristics of amine modifications that either enhance or detract from hemocompatibility.

**Figure 6:**
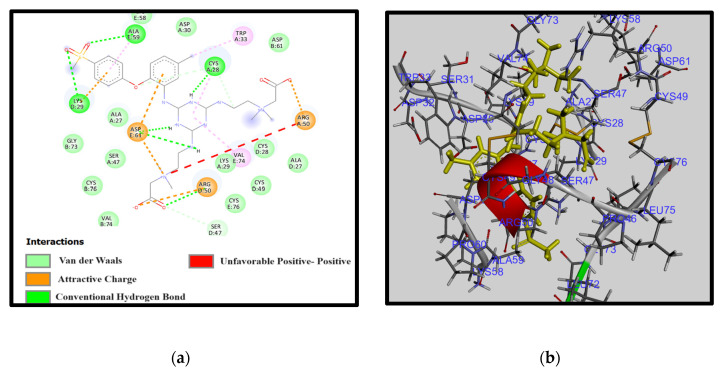
(**a**) The 2D interaction diagrams and (**b**) electrostatic maps of interactions of docking PES-M-TA-CB with FB.

**Figure 7:**
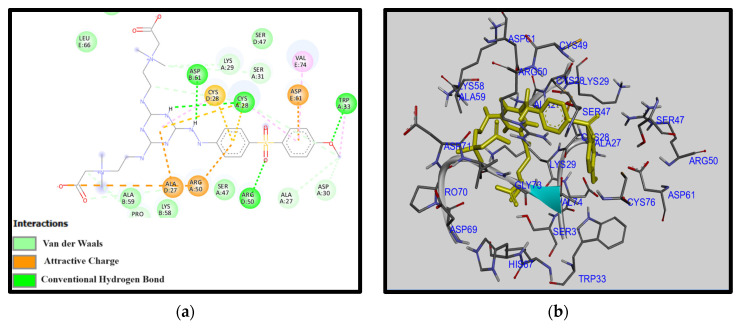
(**a**) Two-dimensional interaction diagrams and (**b**) electrostatic maps of interactions of docking PES-S-TA-CB with FB.

Figure 6 and Figure 7 depict the outcomes of the molecular docking simulations for two distinct amine-modified PES membranes interacting with fibrinogen (FB). Utilizing AutoDock tools and Discovery Studio Visualizer, our analyses revealed significant variations in the optimized structures, interaction patterns, and binding affinities between these amine-modified membranes. These differences highlight the distinct molecular mechanisms through which each amine modification influences the hemocompatibility of PES membranes.

Figure 6 illustrates the middle amine structure, showing a complex network of interactions with fibrinogen. This conformation appears contracted, potentially increasing steric hindrance and electrostatic repulsion, exemplified by the unfavorable positive–positive interactions involving Arg A:50. Such interactions suggest a higher energy state and less stable interaction profile, indicating potentially lower hemocompatibility. Unfavorable interactions can lead to increased protein adsorption on biomaterial surfaces, promoting fouling, which compromises material performance and longevity.

In contrast, Figure 7 portrays the side amine structure in a more linear conformation. This distinct orientation fosters a different interaction pattern, characterized by hydrogen bonds with amino acids Asp B:61, Cys A:28, Arg D:50, and Trp A:33, compared to the middle amine structure, which engages in hydrogen bonding with Ala E:59, Lys D:29, and Cys A:28. The presence of a robust hydrogen-bonding network in the side amine structure acts as a fortified barrier against protein adhesion.

The absence of unfavorable electrostatic interactions in the side amine structure, combined with its formidable hydrogen-bonding scaffold, enhances its hemocompatibility by minimizing indiscriminate protein adsorption, which could lead to material fouling. This structural advantage underscores the potential of side amine modifications in enhancing the performance and longevity of biomaterials in contact with blood. In conclusion, molecular docking analyses provide valuable insights into how amine modifications influence the hemocompatibility of PES membranes. By elucidating specific interaction mechanisms and structural features, these computational tools contribute crucially to the design and optimization of biomaterials for biomedical applications, ensuring safer and more effective clinical outcomes.

### 3.2. Evaluating Water Stability in Membrane Models

The relationship between membrane hemocompatibility and water mobility is pivotal in advancing hemodialysis (HD) technologies. Traditionally, improved hemocompatibility was linked to increased hydrophilicity, indicated by lower contact angles and surface free energy, suggesting better antithrombogenic and anticoagulation properties. However, recent studies, including our team’s work, have shifted focus towards understanding the role of water dynamics within the membrane. The presence of intermediate water molecules—neither fully mobile like free water nor static like non-freezable water—has been found crucial in enhancing hemocompatibility [18]. These intermediate water layers act as protective barriers, influencing the interaction with proteins and uremic metabolites, thereby improving the membrane’s performance.

**Figure 8:**
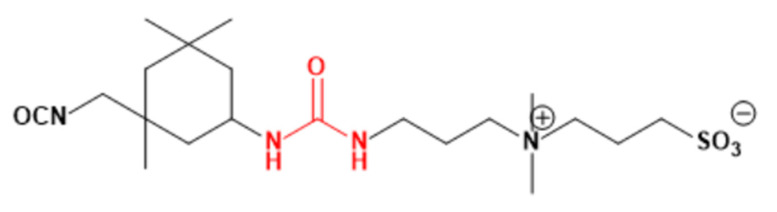
Urea attached to zwitterionic structure as a modifier for ethylene/vinyl alcohol (EVOH) membrane; red part represents urea. Chemical structure created under patent by Jianxiu, 2020 [30].

The development of TA-CB membranes is rooted in the quest for hemocompatible structures that integrate urea within a zwitterionic polymer framework. Recently patented, the structure illustrated in Figure 8 has emerged as a promising membrane modifier, demonstrating effective biocompatibility [30]. Each component of this innovative structure plays a critical role: the uremic metabolite-like segment enhances super-hydrophilicity, facilitating robust water adsorption, while the zwitterionic (ZW) part acts as a protective shield against protein fouling on the membrane surface. This dual functionality not only safeguards the attached uremic metabolite from unwanted interactions with serum proteins but also ensures a stable, biocompatible interface crucial for hemodialysis applications.

**Figure 9:**
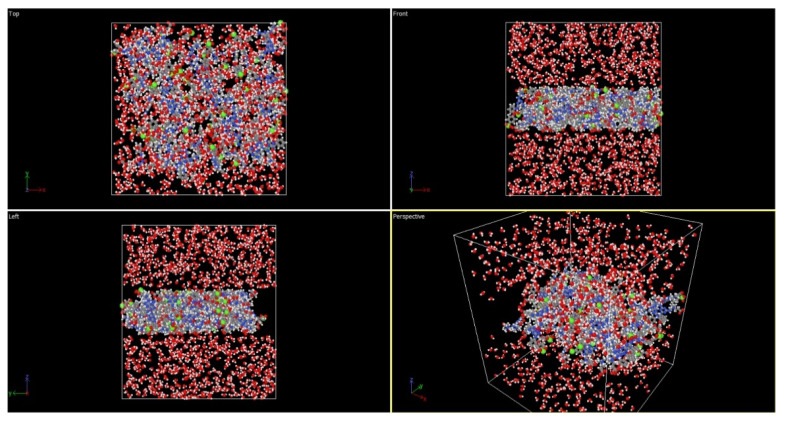
MD capture of hydrated PES-M-TA-CB box.

**Figure 10:**
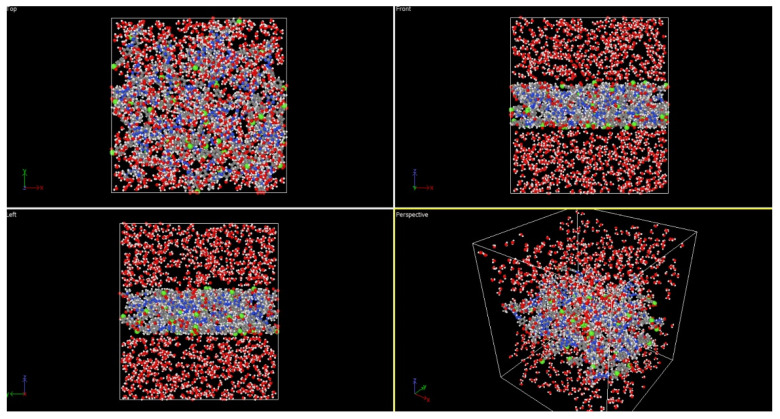
MD capture of hydrated PES-S-TA-CB box.

Inspired by guanidine-like structures characterized by a carbon atom attached to three nitrogen atoms, TA-CB structures are strategically designed to incorporate cyclic nitrogen-containing moieties. Previous studies have highlighted the advantages of cyclic nitrogen structures in enhancing water absorption and creating additional surface area compared to their linear counterparts. Building upon these insights, the proposed TA-CB membranes leverage cyclic nitrogen structures synergistically with a zwitterionic carboxybetaine component. MD simulations, depicted in Figure 9 and Figure 10, provide visual confirmation of the structural integrity and behavior under simulated physiological conditions.

In the visual presentation of our molecular dynamics simulations, we selected a black background for our figures to enhance clarity and effectiveness. The black background provides a stark contrast against the colors used for hydrogen (white) and oxygen (red) atoms in the water box, making these atoms more distinct and easier to identify. Additionally, it aids in differentiating the various colors used to represent membrane ligands, thereby improving readability and interpretation. Furthermore, the black background reduces glare and highlights molecular structures, which is particularly advantageous in high-resolution images, as it emphasizes details and enhances the overall visual appeal.

**Figure 11:**
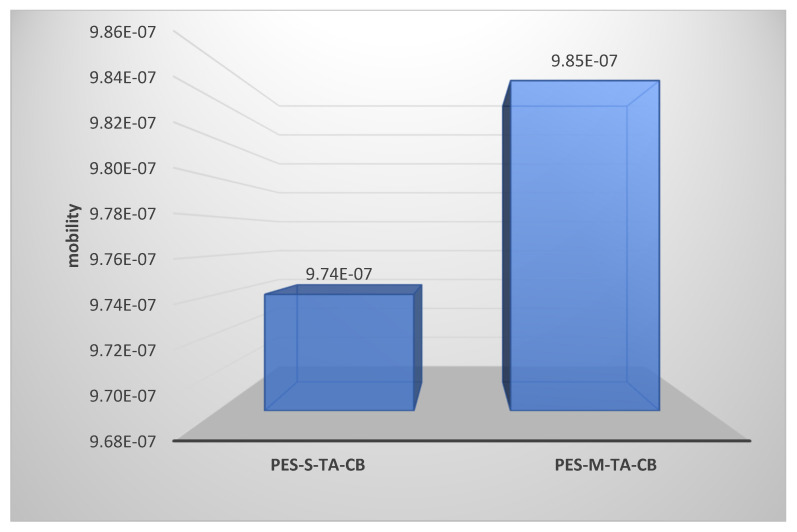
Mobility values for modeled hydrated membranes.

The hemocompatibility of hemodialysis membranes critically depends on the stability of the water molecules adsorbed onto their surfaces [16,25,31]. A stable hydration layer functions as a protective barrier, minimizing interactions between serum proteins and the polymer surface. Figure 11 presents the detailed mobility values obtained from the MD simulations of the PES-S-TA-CB and PES-M-TA-CB structures. Notably, middle amine functionalization resulted in a mobility value of 9.85 × 10^−7^, whereas side amine functionalization via aminolysis slightly reduced this value to 9.74 × 10^−7^, indicative of enhanced surface stability and reduced surface protein interactions.

The strategic incorporation of nitrogen atoms within the backbone of the immobilized structure, particularly evident in the PES-S-TA-CB configurations, promotes greater association with water molecules, thereby enhancing stability and reducing surface mobility (lower mobility value). In an optimally functionalized surface devoid of material aggregation, the S-TA-CB structures create microenvironments conducive to sustained water presence and efficient hemodialysis performance. This design approach not only underscores the importance of structural integrity but also highlights the potential for tailored membrane solutions that optimize biocompatibility in medical settings.

In conclusion, the expanded discussion elucidates the intricate design principles, molecular dynamics insights, and the pivotal role of water stability in advancing the hemocompatibility of TA-CB-functionalized membranes. By leveraging cyclic nitrogen structures and zwitterionic components, TA-CB membranes offer a promising avenue for enhancing biocompatibility and performance in hemodialysis applications, promising safer and more effective medical treatment.

### 3.3. Integrating Computational Studies with Hemocompatibility

Hemocompatibility is a critical factor in the design and performance of hemodialysis membranes, directly influencing patient safety and treatment efficacy. Effective membranes must minimize adverse interactions with blood components to reduce risks such as thrombosis and inflammation. Our study aimed to enhance hemocompatibility by evaluating the impact of different amine modifications on PES membranes, specifically focusing on their interactions with human serum fibrinogen and water stability.

Our molecular docking analyses revealed significant differences between the side and middle amine functionalizations of PES membranes in terms of their interactions with fibrinogen. The side amine modification exhibited a more favorable interaction profile, characterized by a lower binding affinity and fewer unfavorable electrostatic interactions. This suggests that side-amine-functionalized membranes are less likely to trigger adverse responses, such as clot formation, which is crucial for reducing thrombosis risk and improving patient safety in hemodialysis applications. In contrast, the middle amine functionalization demonstrated less-favorable binding interactions, including increased electrostatic repulsion and steric hindrance. These factors contribute to higher protein adsorption and potential fouling, which could compromise membrane performance and longevity. Our results align with the existing literature, which highlights the importance of optimizing functionalization strategies to enhance blood compatibility and reduce clottingrisks [32,33,34]. By demonstrating that side amine modifications offer superior hemocompatibility, our study advances the development of hemodialysis membranes and supports the goal of improving patient outcomes.

Water stability plays a pivotal role in understanding membrane performance and its relationship with hemocompatibility. Our molecular dynamics simulations revealed that side-amine-functionalized membranes exhibited reduced water mobility compared to middle-amine-functionalized membranes. This enhanced water stability is associated with decreased protein adsorption and improved membrane performance, as a stable hydration layer acts as a protective barrier against fouling. Comparative studies have shown that improved water stability in hemodialysis membranes correlates with better patient outcomes, including a reduced incidence of inflammation [12,13,18,30]. The reduced water mobility observed in side-amine-functionalized membranes suggests a more stable and biocompatible interface, which is beneficial for effective hemodialysis. This finding underscores the importance of water dynamics in membrane design and highlights the potential of side amine modifications to enhance hemocompatibility and overall treatment efficacy.

Our findings are consistent with recent research emphasizing the advantages of optimized membrane functionalization in improving blood compatibility and reducing clotting risks [35,36,37,38]. Recently, researchers have proposed that water interacting with hydrophilic surfaces can exist in three distinct structures: free water, which freezes at 0 °C; non-freezable water, which remains unfrozen even at extremely low temperatures due to close associations with polymeric structures; and intermediate water, which exhibits mobility between that of free and non-freezable water. This stable water layer was suggested to improve hemocompatibility by reducing clotting risks [39]. Studies also indicate that fibrinogen interactions and adsorption on membranes can have several adverse effects on patients receiving dialysis [40]. By demonstrating that side amine modifications outperform middle amine modifications in both interaction with fibrinogen and water stability, our study supports and extends these conclusions. These results contribute to the ongoing efforts to refine hemodialysis technologies and improve patient outcomes, providing valuable insights for future membrane development.

While our computational analyses offer a solid foundation for improving membrane hemocompatibility, experimental validation is essential to confirm these findings in clinical settings. Future research should focus on synthesizing and testing these modified membranes to assess their performance and effectiveness in real-world hemodialysis scenarios. Additionally, exploring other functionalization strategies and their impact on membrane properties could further enhance the development of optimized hemodialysis membranes. In conclusion, our study highlights the significant advantages of side amine functionalization in enhancing the hemocompatibility of PES membranes. By demonstrating improved interactions with fibrinogen and superior water stability, we provide crucial insights into designing safer and more effective hemodialysis membranes. These advancements hold promise for improving patient outcomes and advancing hemodialysis technology, paving the way for more effective and safer treatments in the future.

## 4. Conclusions

This paper proposes two distinct methods for the immobilization of zwitterionic materials on PES membranes, highlighting differences in amine functionalization approaches as the initial step of immobilization. Our investigation, guided by water mobility values, demonstrates that the side amine functionalization approach results in lower mobility values, indicative of higher stability. This stable hydrated system fosters a protective layer on the membrane surface, enhancing its hemocompatibility profile. Molecular docking analyses further corroborate these findings, revealing that the side amine structure exhibits an interaction affinity energy of −7.6 with fibrinogen (FB) compared to −8.2 for the middle amine structure. This difference underscores the superior hemocompatibility of the side amine modification. Additionally, the side amine structure forms hydrogen bonds with four amino acids, contrasting with the middle amine structure’s interaction with only three amino acids. This augmented hydrogen-bonding network strengthens the membrane’s resistance to protein adhesion, further supporting its hemocompatible nature.

Our findings significantly contribute to advancing membrane compatibility, potentially mitigating complications during dialysis procedures. Moving forward, our study suggests promising avenues for future research, particularly in the experimental validation of these computational predictions. The practical implications are substantial, offering a pathway towards developing more hemocompatible dialysis membranes. Exploring alternative zwitterionization techniques may unveil new strategies to further enhance the efficiency and biocompatibility of PES membranes.

While computational models provide valuable insights, it is essential to acknowledge their inherent limitations in fully capturing the complexities of biological interactions and real-world scenarios. Variations in individual patient responses, dynamic physiological conditions, and unforeseen interactions with other biomolecules can significantly influence the practical performance of modified membranes. Therefore, rigorous experimental validation is imperative to validate the applicability and reliability of these computational predictions [36,37]. Moreover, considerations such as the feasibility of manufacturing these modified membranes, addressing regulatory requirements, and assessing scalability and cost-effectiveness are pivotal for successful clinical implementation of new membrane technologies.

In conclusion, the side amine functionalization approach via aminolysis emerges as a promising strategy for enhancing the hemocompatibility of PES membranes. By integrating computational insights with experimental validation and addressing practical considerations, we aim to propel the development of next-generation dialysis membranes that improve patient outcomes and quality of life.

## Figures and Tables

**Table 1:** Bond parameters.

Bonds	R_0_ (Å)
C-C	1.53
C-H	0.98
N-C	1.46
O-C	1.42
S-C	1.8
N-H	1.02
O-H	0.98
S-S	2.07

**Table 2:** Angle parameters.

Central Atom	Angle Θ_0_ (Degrees)
Carbon	109.47
Hydrogen	180
Nitrogen	106.7
Sulfur	92.1
Oxygen	104.51

**Table 3:** Non-bond parameters.

Atom	Ε (kcal/mol)	Σ (A)
C	0.0238	3.473
H	0.0038	2.846
N	0.0194	3.263
O	0.0239	3.033
S	0.0860	3.590

**Table 4:** Chemical structures of HD membrane modifiers and uremic metabolites.

No.	Name	Functionalization Method	Structure	Simulation Box Size	Number of Atoms	Number of Water Molecules
1	PES-M-TA-CB	Middle amine	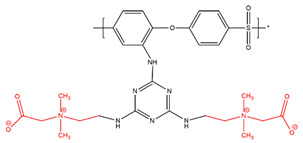	70 × 70 × 78	6900	1000
2	PES-S-TA-CB	Side amine	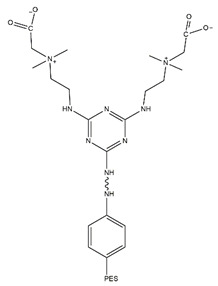	70 × 70 × 78	6950	1000

## Data Availability

The raw/processed molecular docking data required to reproduce these findings cannot be shared at this time, as they are critical to ongoing research.
